# Trends and determinants of HIV transmission among men who inject drugs in the Pokhara Valley, Nepal: analysis of cross-sectional studies

**DOI:** 10.1186/s12889-021-10331-9

**Published:** 2021-02-02

**Authors:** Sam Hogan, Andrew Page, Felix Ogbo, Sameer Dixit, Rajesh Man Rajbhandari, Bir Rawal, Keshab Deuba

**Affiliations:** 1 Translational Health Research Institute, Western Sydney University Sydney, Locked Bag 1797, Penrith, NSW 2751 Australia; 2 School of Medicine, Western Sydney University, Sydney, New South Wales Australia; 3 Center For Molecular Dynamics, Kathmandu, Nepal; 4 National Center for AIDS and STD Control, Kathmandu, Nepal; 5 Department of Global Public Health, Karolinska Institutet, Stockholm, Sweden; 6 National Centre for AIDS & STD Control/ Global Fund Programs, Kathmandu, Nepal

**Keywords:** Trends, Determinants, Drug use, Behavioural, Male, Nepal

## Abstract

**Background:**

HIV is a major public health issue around the world, especially in developing countries. Although the overall prevalence of HIV in Nepal is relatively low, there are specific sub-populations where the prevalence is far higher than the national average. One of these sub-groups is male people who inject drugs (male PWIDs). In order to understand the reasons for the differences in prevalence, a series of socio-demographic, behavioural and knowledge-based risk factors need to be assessed.

**Methods:**

The study used a series of 7 cross-sectional survey datasets from Pokhara (Nepal), collected between 2003 and 2017 (*N* = 2235) to investigate trends in HIV prevalence among male PWIDs by socio-demographic and behavioural and knowledge-based risk factors. A series of logistic regression models were conducted to investigate the association between study factors and HIV.

**Results:**

HIV prevalence decreased from the levels seen in 2003 (22.0%) and 2005 (21.7%), with the lowest prevalence recorded in 2015 (2.6%), however prevalence has increased in the most recent period (4.9%). A lower risk of HIV was associated with younger age (<=24 years compared to > 24 years, OR = 0.17, 95% CI = 0.10–0.31), being married (OR = 1.91, 95% CI = 1.25–3.02) and shorter duration of drug use (<=4 years compared to > 4 years, OR = 0.16, 95% CI = 0.09–0.29). A higher risk of HIV was associated with low (compared to secondary or higher) education level (OR = 2.76, 95% CI = 1.75–4.36), a lack of addiction treatment (OR = 2.59, 95% CI = 1.64–4.08), and recent use of unsterilized injection equipment (OR = 2.22, 95% CI = 1.20–4.11).

**Conclusion:**

The prevalence of HIV in male PWIDs in Pokhara has been variable, but overall has reduced in recent years to 2.6% before increasing in 2017 to 4.9%. The main determinants which increase the risk of HIV among male PWIDs in Pokhara are low education level, a lack of treatment for drug addiction and the recent use of unsterilised equipment. Each of these indicate the need to improve addiction treatment and education programs for intra-venous drug use to aid this key population in avoiding risk-taking behaviours.

**Supplementary Information:**

The online version contains supplementary material available at 10.1186/s12889-021-10331-9.

## Background

The spread of human immunodeficiency virus (HIV) among key populations is an important health issue around the world [1]. Several factors have been shown to increased HIV transmission in many communities worldwide. These include safe sex practices, needle sharing habits, intravenous drug use, poor equipment cleaning practices and alcohol consumption [2–4]. Stigma, discrimination and misinformation also play important roles in HIV transmission as they prevent correct dissemination of appropriate information and implementation of preventive methods [5, 6].

In Nepal, there are many key populations in some specific locations (the Pokhara Valley and Kathmandu), with a high number of factors which increase the risk of disease transmission like increased economic activities, unsafe sexual practices, and increased levels of drug use [7–16]. These key populations include people who inject drugs, female sex workers, men who have sex with men and labour migrants [17]. These groups are at particularly high risk of contracting HIV and other blood borne diseases such as hepatitis C infection [18]. Without routine testing being readily available and accessible or community-based education on the risks of HIV transmission, there is an increased likelihood of HIV disease burden throughout the community [13].

Previous studies from Nepal have determined the incidence and prevalence of HIV among the key populations to provide relevant data for targeted interventions [8, 11, 12, 14]. However, those studies focused on specific geographical areas, such as Kathmandu or administrative Development regions of the country, and have not focussed on others where there are higher levels of risks and determinants of HIV transmission, such as the Pokhara valley [17]. The Pokhara valley contains the capital city of Gandaki Province, and is considered to be the tourism capital of Nepal due to its proximity to the Himalayas. The city of Pokhara forms a major tourism and manufacturing hub within Nepal and had an estimated population of approximately 523,000 in 2020. As this city is a major population centre, and the 2nd largest in Nepal, it is important to evaluate the risk factors of HIV within this setting.

While Nepal is considered to be a low prevalence population [19], monitoring and evaluating the HIV epidemiology in specific high-risk locations is still an important public health priority [17]. Public health surveillance programs for HIV has also been recognised as an issue in Nepal [19].

In a recent paper, it was determined that condom adherence is more strongly associated with socio-demographic features, such as socio-economic status, than factors such as education level or knowledge of HIV [20]. This could indicate that sexual health education programs are not currently working as effectively as necessary. Caste and ethnicity also act as major determinants of overall health literacy and knowledge of preventative measures [21]. Injecting drugs at any point within a year has been shown to be a risk factor for non-utilisation of HIV testing centres [22], meaning that the study population are less likely to access these HIV preventative measures than other key populations.

There has been previous descriptive research in the Kathmandu Valley in Nepal into key risk factors and prevalence trends [7–9, 17], however, more detailed analytic approaches to investigate socio-demographic and behavioural factors are currently lacking for this setting. Previous behavioural studies have also shown that there are multiple risk factors that act as determinants for HIV and other sexually transmitted diseases within other areas of Nepal [10, 11], such as social class and education level [21]. Accordingly, this study aimed to investigate trends and the associations between socio-demographic, behavioural and knowledge-based factors and HIV transmission among male injection drug users (IDUs) in the Pokhara valley, Nepal from 2003 to 2017. This would provide a more detailed perspective of HIV information among this cohort to inform policy and prevention strategies.

## Methods

The Integrated Biological Behavioural Surveys (IBBS) are a series of surveys taken once every two years in Nepal, beginning in 2003. There have been 7 iterations of this survey. The surveys are intended to track the prevalence of various diseases among targeted populations, in this case men who inject drugs. In addition to assessing disease prevalence, the surveys recorded demographic, behavioural and social factors as part of the surveillance of HIV in Nepal [17]. The key populations that the IBBS focus on are people who inject drugs (PWIDs), labour migrants, sex workers spouses and men who have sex with men [17].

### Study sample

This study uses a series of behavioural surveys taken between 2003 and 2011 and 2015–2017. The total number of participants across this period was 2235 men over the age of 16 who had been injecting drugs for a period of at least three months. Participants were recruited using respondent driven sampling methods, where seeds from the specific target population of men who inject drugs were selected. A full description of the selection and sampling methods are available in the IBBS [17]. The 2003, 2005, 2007 and 2009 surveys all had 300 participants, while the 2011, 2015 and 2017 surveys each had 345.

**Fig. 1:**
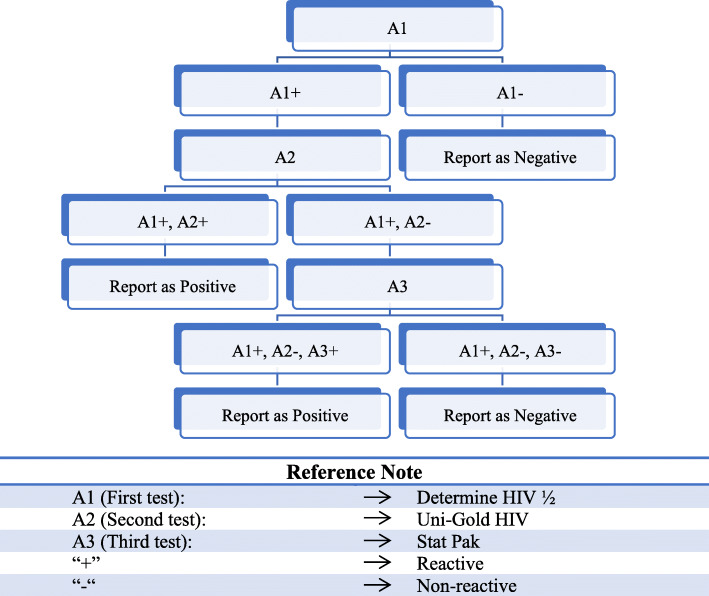
Testing cascade for the rapid HIV diagnostic tests prior to 2017. Source: IBBS 2015 Report

Face-to-face interviews were performed with participants’ answers being recorded on a set questionnaire, with the 2017 survey being performed on tablets. The IBBS surveys recorded a wide array of variables, a selection of which were used in the present study (a full list of which is available at the end of each IBBS survey). HIV testing was performed using the Determine HIV 1/2 test (manufactured by Abbot, Japan), which detects the presence of antibodies against HIV. If this first test produced a positive result, a second and third test were used for confirmation. These tests were Uni-Gold (Trinity Biotech, Ireland) and Stat-Pak HIV 1/2 (Chembio Diagnostics). The third test was only used if there was a disagreement between the first two tests used. If the first test was negative, then no further testing was done. The full testing algorithm is available in the IBBS reports [17], and also shown in Fig. 1. In the 2017 IBBS study, the WHO HIV testing strategy was used. This involved using three consecutive reactive tests as the basis for HIV positive diagnoses. Stat-Pak HIV1/2 was the mandatory kit to confirm any HIV positive diagnoses.

### Outcome

The main outcome of interest in this study was HIV infection. This was defined by use of two rapid detection kits and collected from participants by testing blood samples. The tests were used in a cascading series, where if the first test returned a positive result, the second test would be used to confirm or contradict this result [17]. Cases were determined to be HIV positive only if all three tests showed the blood sample to be reactive. The testing cascade differed in the earlier surveys (2003–2011) so that positives were counted either if the first and second tests were both positive. This difference was due to the different testing kits used from 2003 to 11.

### Risk factors

Socio-demographic factors included age, education, and marital status, which were all defined as binary variables to maximise the size of comparator groups given the small sample size available at each time-period. Age was defined as being either “above” or “below” the median age. Similarly, education status was defined as being “primary or lower school education” and “secondary or higher school education”, while marital status was categorised as a “married” or “not married”. Living situation, which was defined as living “with” or “without” a sexual partner was also included in socio-demographic factors.

Behavioural factors included having undergone treatment for drug addiction, being tested for HIV, visiting an HIV testing and counselling centre, duration of intravenous drug use, use of a female sex worker, needle sharing behaviours, alcohol intake, condom use, use of unsterilized injecting equipment and the age at which drugs were first injected. Most of these factors were binary “yes” or “no” variables. Duration of drug use and age at first injection were defined as being “above” or “below” the median, while alcohol intake was broken into “everyday”, “sometimes” and “never”.

### Participant characteristics

Overall, the average age of male PWIDs interviewed between 2003 and 2017 increased. The lowest average age occurred in the 2003 survey (average age of 23.4 years), and generally increased with each iteration of the survey. Average age decreased slightly between 2007 and 2009 (24.8 to 24.5 years), before increasing in 2011 (24.9 years). The highest average age occurred in the 2017 (28.2 years).

The education status of the respondents increased every year between 2003 and 2015, before decreasing in 2017. The highest completed grade of school on average in the surveys occurred in 2011 and 2015 (Grade 9). The average highest completed grade in 2003 was Grade 7, before increasing to Grade 8 in all other years.

Factors relating to knowledge of HIV included knowing someone with HIV/AIDS, having met and discussed HIV with a peer/community educator (PE/CE), and knowledge of where to access antiretroviral therapy.

A summary of participant characteristics is provided in Supplementary Table 1.

### Analysis

Prevalence estimates and 95% confidence intervals for HIV were examined over the period spanning 2007–2017, stratified by each of the study variables described above.

A separate series of logistic regression models that were restricted to the period 2007 to 2017 were also conducted. Models were restricted to this period because the prevalence numbers expressed in 2003 and 2005 were likely substantially affected by measurement bias. This was due to overestimation of the positive results, which may have been due to a change in the HIV testing regime and the definition of a positive case. In these years, HIV testing was performed using two rapid tests (“Capillus” and “Determine”), with disagreements being resolved with the use of “Uni-Gold TM”.

Univariable and multivariable logistic regression models were conducted to investigate the association between the socio-demographic, behavioural and knowledge factors described above and HIV status. Models were aggregated over the period 2007–2017. Additionally, single years were investigated to evaluate the magnitude of change of relative associations between different time points. Multivariable models adjusted for the potential confounding factors of age, education level and marital status. Multivariable models adjusted for the key potential confounding factors of age, education level and marital status. Knowledge, behavioural and health service determinants were considered for inclusion in multivariable models, however given small numbers of cases within strata for some variables and the cross-sectional data used, the putative causal directions between these variables could not be clearly specified, therefore a minimal set of socio-demographic factors were adjusted for. All analyses were conducted in R Studio using the glm and confint.lm functions, which produced the estimates for odds ratios and 95% confidence intervals for each of these models.

## Results

HIV prevalence decreased over the study period 2003 to 2015 from 22 to 2.6% before rising again in 2017 to 4.9% (Table 1). The lowest prevalence for HIV in the study populations occurred in 2015 (2.6 95% CI 1.2–4.9%), however prevalence has increased in the most recent period in 2017 numbers (4.9, 95% CI 2.9–7.8%). There was a decrease in every survey prior to 2017, with the largest decrease occurring between 2005 (21, 95% CI 17.1–26.8%) and 2007 (8.6, 95% CI 5.7–12.4%). HIV prevalence was higher among participants who were above the median age in all years, and was also lower in participants who had achieved secondary education or higher in all years except for 2003 (Table 1.) Having injected with a used syringe in the previous week had a higher prevalence of HIV in all years except for 2015 (Table 2). HIV prevalence was higher among participants who had received treatment for drug addiction in all years (Table 3). Individuals who knew others with HIV/AIDS and knew where to receive ART also had a higher prevalence of HIV (Table 3.).

**Table 1:** Prevalence of HIV based on selected socio-demographic characteristics of IBBS participants, Pokhara Valley (Nepal)

	2003			2005			2007			2009			2011			2015			2017		
	n(a)	N(b)	% (95% CI)	n	N	% (95% CI)	n	N	% (95% CI)	n	N	% (95% CI)	n	N	% (95% CI)	n	N	% (95% CI)	n	N	% (95% CI)
*Age*																					
Below median	21	161	13.0 (8.3–19.2)	19	164	11.6 (7.1–17.5)	6	163	3.7 (1.4–7.8)	4	167	2.4 (0.7–6.0)	3	186	1.6 (0.3–4.6)	0	180	0.0 (0.0–2.0)	2	185	1.1 (0.1–3.9)
Above median	45	139	32.4 (24.7–40.8)	46	136	33.8 (25.9–42.4)	20	137	14.6 (9.2–21.6)	13	133	9.8 (5.3–16.1)	13	159	8.2 (4.4–13.6)	9	165	5.5 (2.5–10.1)	15	160	9.4 (5.3–15.0)
*Educational achievement*																					
Primary or lower	20	100	20.0 (12.7–29.2)	28	114	24.6 (17.0–33.5)	14	94	14.9 (8.4–23.7)	11	90	12.2 (6.3–20.8)	5	62	8.1 (2.7–17.8)	3	62	4.8 (1.0–13.5)	4	49	8.2 (2.3–19.6)
Secondary or higher	46	200	23.0 (17.4–29.5)	37	186	19.9 (14.4–26.4)	12	206	5.8 (3.0–10.0)	6	210	2.9 (1.1–6.1)	11	283	3.9 (2.0–6.8)	6	283	2.1 (0.8–4.6)	13	296	4.4 (2.4–7.4)
*Marital status*																					
Married	39	218	17.9 (13.0–23.6)	39	213	18.3 (13.4–24.2)	10	105	9.5 (4.7–16.8)	6	96	6.3 (2.3–13.1)	6	100	6.0 (2.2–12.6)	8	109	7.3 (3.2–14.0)	11	129	8.5 (4.3–14.7)
Not married	27	82	32.9 (22.9–44.2)	26	87	29.9 (20.5–40.6)	16	195	8.2 (4.8–13.0)	11	204	5.4 (2.7–9.4)	10	245	4.1 (2.0–7.4)	1	236	0.4 (0.0–2.3)	6	216	2.8 (1.0–5.9)
*Living with sexual partner*																					
Yes	23	80	28.8 (19.2–40.0)	25	85	29.4 (20.0–40.3)	8	99	8.1 (3.6–15.3)	5	88	5.7 (1.9–12.8)	6	96	6.3 (2.3–13.1)	5	83	6.0 (2.0–13.5)	5	61	8.2 (2.7–18.1)
No	39	220	17.7 (12.9–23.4)	40	215	18.6 (13.6–24.5)	18	201	9.0 (5.4–13.8)	12	212	5.7 (3.0–9.7)	10	249	4.0 (1.9–7.3)	4	262	1.5 (0.4–3.9)	12	284	4.2 (2.2–7.3)
Total	66	300	22.0 (17.4–27.1)	65	300	21.7 (17.1–26.7)	26	300	8.7 (5.7–12.4)	17	300	5.7 (3.3–8.9)	16	345	11.3 (2.7–7.4)	9	345	2.6 (1.2–4.9)	17	345	4.9 (2.9–7.8)

*n* Number of HIV cases, *N* Total number of participants, *IBBS* Integrated Biological Behavioural Surveys

**Table 2:** Prevalence of HIV based on selected drug injecting behaviours of IBBS participants, Pokhara Valley (Nepal)

	2003			2005			2007			2009			2011			2015			2017		
	n(a)	N(b)	% (95% CI)	n	N	% (95% CI)	n	N	% (95% CI)	n	N	% (95% CI)	n	N	% (95% CI)	n	N	% (95% CI)	n	N	% (95% CI)
*Injected on previous day*																					
Yes	55	243	22.6 (17.5–28.4)	46	145	31.7 (24.3–40.0)	23	236	9.7 (6.3–14.3)	17	236	7.2 (4.3–11.3)	2	206	1.0 (0.1–3.5)	1	41	2.4 (0.1–12.9)	3	152	2.0 (0.4–5.7)
No	11	57	19.3 (10.0–31.9)	22	155	14.2 (9.1–20.7)	3	64	4.7 (1.0–13.1)	0	64	0.0 (0.0–5.6)	9	139	6.5 (3.0–11.9)	8	304	2.6 (1.1–5.1)	14	193	7.3 (4.0–11.9)
*Recently used unsterilised needles*																					
Yes	–	–	–	8	37	21.6 (9.8–38.2)	2	20	10.0 (1.2–31.7)	4	28	14.3 (4.0–32.7)	0	6	0.0 (0.0–45.9)	0	34	0.0 (0.0–10.3)	–	–	–
No	–	–	–	18	263	6.8 (4.1–10.6)	15	280	5.4 (3.0–8.7)	12	317	3.8 (2.0–6.5)	7	244	2.9 (1.2–5.8)	16	292	5.5 (3.2–8.7)	–	–	–
*Injected with a used syringe in the past week*																					
Yes	18	63	28.6 (17.9–41.3)	11	44	25.0 (13.2–40.3)	7	25	28.0 (12.1–49.4)	2	16	12.5 (1.6–38.3)	2	8	25.0 (3.2–65.1)	0	1	0.0 (0.0–97.5)	–	–	–
No	48	229	21.0 (15.9–26.8)	46	195	23.6 (17.8–30.2)	19	267	7.1 (4.3–10.9)	15	276	5.4 (3.1–8.8)	11	307	3.6 (1.8–6.3)	3	139	2.2 (0.4–6.2)	–	–	–
Not applicable	0	8	0.0 (0.0–36.9)	8	61	13.1 (5.8–24.2)	0	8	0.0 (0.0–36.9)	0	8	0.0 (0.0–36.9)	3	30	10.0 (2.1–26.5)	3	205	1.5 (0.3–4.2)	–	–	–
*Gave needle to someone else after use in last week*																					
Yes	12	66	18.2 (9.8–29.6)	8	38	21.1 (9.6–37.3)	3	16	18.8 (4.0–45.6)	1	9	11.1 (0.3–48.2)	1	8	12.5 (0.3–52.7)	0	3	0.0 (0.0–70.8)	0	11	0.0 (0.0–28.5)
No	54	226	23.9 (18.5–30.0)	49	201	24.4 (18.6–30.9)	23	276	8.3 (5.4–12.2)	16	283	5.7 (3.3–9.0)	12	307	3.9 (2.0–6.7)	6	137	4.4 (1.6–9.3)	17	333	5.1 (3.0–8.0)
Not applicable	0	8	0.0 (0.0–36.9)	8	61	13.1 (5.8–24.2)	0	8	0.0 (0.0–36.9)	0	8	0.0 (0.0–36.9)	3	30	10.0 (2.1–26.5)	0	205	0.0 (0.0–1.8)	0	1	0.0 (0.0–97.5)
*Used a pre-filled syringe in past week*																					
Yes	5	23	21.7 (7.5–43.7)	5	25	20.0 (6.8–40.7)	4	17	23.5 (6.8–49.9)	1	41	2.4 (0.1–12.9)	1	8	12.5 (0.3–52.7)	0	3	0.0 (0.0–70.8)	1	27	3.7 (0.1–19.0)
No	61	269	22.7 (17.8–28.2)	51	213	23.9 (18.4–30.3)	22	275	8.0 (5.1–11.9)	16	251	6.4 (3.7–10.1)	12	307	3.9 (2.0–6.7)	6	137	4.4 (1.6–9.3)	16	316	5.1 (2.9–8.1)
Not applicable	0	8	0.0 (0.0–36.9)	8	53	15.1 (6.7–27.6)	0	8	0.0 (0.0–36.9)	0	8	0.0 (0.0–36.9)	3	30	10.0 (2.1–26.5)	0	205	0.0 (0.0–1.8)	0	2	0.0 (0.0–84.2)
*Know where to get clean needles*																					
Yes	66	300	22.0 (17.4–27.1)	65	294	22.1 (17.5–27.3)	24	295	8.1 (5.3–11.9)	17	299	5.7 (3.3–8.9)	16	344	4.7 (2.7–7.4)	9	310	2.9 (1.3–5.4)	17	301	5.6 (3.3–8.9)
No	0	0	–	0	6	0.0 (0.0–45.9)	2	5	40.0 (5.3–85.3)	0	1	0.0 (0.0–97.5)	0	1	0.0 (0.0–97.5)	0	35	0.0 (0.0–10.0)	0	44	0.0 (0.0–8.0)
*Average age at first injection*																					
Above median	30	169	17.8 (12.3–24.4)	35	152	23.0 (16.6–30.5)	17	153	11.1 (6.6–17.2)	11	169	6.5 (3.3–11.3)	13	178	7.3 (3.9–12.2)	2	196	1.0 (0.1–3.6)			
Below median	36	131	27.5 (20.0–36.0)	3	148	2.0 (0.4–5.8)	9	147	6.1 (2.8–11.3)	6	131	4.6 (1.7–9.7)	3	167	1.8 (0.4–5.2)	7	149	4.7 (1.9–9.4)	13	164	7.9 (4.3–13.2)
Total	66	300	22.0 (17.4–27.1)	65	300	21.7 (17.1–26.7)	26	300	8.7 (5.7–12.4)	17	300	5.7 (3.3–8.9)	16	345	11.3 (2.7–7.4)	9	345	2.6 (1.2–4.9)	17	345	4.9 (2.9–7.8)

*n* Number of HIV cases, *N* Total number of participants, *IBBS* Integrated Biological Behavioural Surveys

**Table 3:** Prevalence of HIV based on selected health factors and knowledge among IBBS participants, Pokhara Valley (Nepal)

	2003			2005			2007			2009			2011			2015			2017		
	n	N	% (95% CI)	n	N	% (95% CI)	n	N	% (95% CI)	n	N	% (95% CI)	n	N	% (95% CI)	n	N	% (95% CI)	n	N	% (95% CI)
*Received addiction treatment*																					
Yes	27	91	29.7 (20.5–40.2)	34	132	25.8 (18.5–34.1)	16	115	13.9 (8.2–21.6)	8	120	6.7 (2.9–12.7)	11	179	6.1 (3.1–10.7)	6	111	5.4 (2.0–11.4)	9	83	10.8 (5.1–19.6)
No	39	209	18.7 (13.6–24.6)	31	168	18.5 (12.9–25.2)	10	185	5.4 (2.6–9.7)	9	180	5.0 (2.3–9.3)	5	166	3.0 (1.0–6.9)	3	234	1.3 (0.3–3.7)	8	262	3.1 (1.3–5.9)
*HIV test*																					
Yes	28	80	35.0 (24.7–46.5)	47	193	24.4 (18.5–31.0)	15	193	7.8 (4.4–12.5)	10	213	4.7 (2.3–8.5)	14	245	5.7 (3.2–9.4)	7	193	3.6 (1.5–7.3)	15	218	6.9 (3.9–11.1)
No	38	220	17.3 (12.5–22.9)	18	107	16.8 (10.3–25.3)	11	107	10.3 (5.2–17.7)	7	87	8.0 (3.3–15.9)	2	100	2.0 (0.2–7.0)	2	152	1.3 (0.2–4.7)	2	127	1.6 (0.2–5.6)
*Condom Use*																					
Always	5	8	62.5 (24.5–91.5)	7	11	63.6 (30.8–89.1)	2	14	14.3 (1.8–42.8)	1	6	16.7 (0.4–64.1)	2	5	40.0 (5.3–85.3)	1	6	16.7 (0.4–64.1)	–	–	–
Sometimes	8	32	25.0 (11.5–43.4)	13	40	32.5 (18.6–49.1)	3	43	7.0 (1.5–19.1)	4	51	7.8 (2.2–18.9)	3	51	5.9 (1.2–16.2)	5	31	16.1 (5.5–33.7)	–	–	–
Never	13	46	28.3 (16.0–43.5)	7	38	18.4 (7.7–34.3)	3	42	7.1 (1.5–19.5)	2	39	5.1 (0.6–17.3)	0	42	0.0 (0.0–8.4)	1	67	1.5 (0.0–8.0)	–	–	–
Not applicable	40	214	18.7 (13.7–24.6)	38	211	18.0 (13.1–23.9)	18	201	9.0 (5.4–13.8)	10	204	4.9 (2.4–8.8)	11	247	4.5 (2.2–7.8)	2	241	0.8 (0.1–3.0)	–	–	–
*Duration of ID use*																					
Below median	23	196	11.7 (7.6–17.1)	14	176	8.0 (4.4–13.0)	6	175	3.4 (1.3–7.3)	6	194	3.1 (1.1–6.6)	3	232	1.3 (0.3–3.7)	1	216	0.5 (0.0–2.6)	2	183	1.1 (0.1–3.9)
Above median	43	104	41.3 (31.8–51.4)	51	124	41.1 (32.4–50.3)	20	125	16.0 (10.1–23.6)	11	106	10.4 (5.3–17.8)	13	113	11.5 (6.3–18.9)	8	129	6.2 (2.7–11.9)	15	162	9.3 (5.3–14.8)
*Used FSW in past year*																					
Yes	13	99	13.1 (7.2–21.4)	18	121	14.9 (9.1–22.5)	12	139	8.6 (4.5–14.6)	3	105	2.9 (0.6–8.1)	6	126	4.8 (1.8–10.1)	4	96	4.2 (1.1–10.3)	6	69	8.7 (3.3–18.0)
No	47	181	26.0 (19.7–33.0)	43	167	25.7 (19.3–33.1)	14	150	9.3 (5.2–15.2)	12	168	7.1 (3.7–12.1)	9	210	4.3 (2.0–8.0)	5	229	2.2 (0.7–5.0)	11	267	4.1 (2.1–7.3)
*Drinking Alcohol*																					
Every day	37	127	29.1 (21.4–37.9)	45	170	26.5 (20.0–33.8)	12	106	11.3 (6.0–18.9)	10	101	9.9 (4.9–17.5)	6	88	6.8 (2.5–14.3)	2	59	3.4 (0.4–11.7)	2	56	3.6 (0.4–12.3)
Sometimes	21	126	16.7 (10.6–24.3)	12	96	12.5 (6.6–20.8)	9	141	6.4 (3.0–11.8)	3	136	2.2 (0.5–6.3)	7	186	3.8 (1.5–7.6)	5	201	2.5 (0.8–5.7)	7	163	4.3 (1.7–8.6)
Never	8	47	17.0 (7.6–30.8)	8	26	30.8 (14.3–51.8)	5	53	9.4 (3.1–20.7)	4	63	6.3 (1.8–15.5)	3	68	4.4 (0.9–12.4)	2	85	2.4 (0.3–8.2)	8	126	6.3 (2.8–12.1)
*Know anyone with HIV/AIDS*																					
Yes	46	194	23.7 (17.9–30.3)	56	240	23.3 (18.1–29.2)	24	233	10.3 (6.7–14.9)	17	223	7.6 (4.5–11.9)	13	245	5.3 (2.9–8.9)	8	229	3.5 (1.5–6.8)	–	–	–
No	19	97	19.6 (12.2–28.9)	5	49	10.2 (3.4–22.2)	2	69	2.9 (0.4–10.1)	0	77	0.0 (0.0–4.7)	3	100	3.0 (0.6–8.5)	1	116	0.9 (0.0–4.7)	–	–	–
Unsure	2	9	22.2 (2.8–60.0)	4	11	36.4 (10.9–69.2)	0	0	–	0	0	–	0	0	–	0	0	–	–	–	–
*Discussed with PE*/*OE/CM/CE*																					
Yes	–	–	–	16	202	7.9 (4.6–12.5)	15	257	5.8 (3.3–9.4)	16	295	5.4 (3.1–8.7)	5	72	6.9 (2.3–15.5)	8	93	8.6 (3.8–16.2)	–	–	–
No	–	–	–	10	98	10.2 (5.0–18.0)	2	43	4.7 (0.6–15.8)	0	50	0.0 (0.0–7.1)	4	273	1.5 (0.4–3.7)	9	252	3.6 (1.6–6.7)	–	–	–
*Knowledge of ART*																					
Yes	–	–	–	15	89	16.9 (9.8–26.3)	6	66	9.1 (3.4–18.7)	8	101	7.9 (3.5–15.0)	9	127	7.1 (3.3–13.0)	16	98	16.3 (9.6–25.2)	–	–	–
No	–	–	–	11	211	5.2 (2.6–9.1)	11	234	4.7 (2.4–8.3)	8	244	3.3 (1.4–6.4)	0	209	0.0 (0.0–1.7)	1	246	0.4 (0.0–2.2)	–	–	–
Total	66	300	22.0 (17.4–27.1)	65	300	21.7 (17.1–26.7)	26	300	8.7 (5.7–12.4)	17	300	5.7 (3.3–8.9)	16	345	11.3 (2.7–7.4)	9	345	2.6 (1.2–4.9)	17	345	4.9 (2.9–7.8)

*n* Number of HIV cases, *N* Total number of participants, *IBBS* Integrated Biological Behavioural Surveys, *FSW* Female Sex Worker, *HIV/AIDS* Human Immunodeficiency virus/ Acquired Immune Deficiency Syndrome, *PE/OE/CM/CE* Peer Educators, Outreach Educators, Community Motivators/Mobilisers, Community Educators, *ART* Antiretroviral therapy

Lower risk of HIV was associated with an education level of secondary school or higher (OR = 0.36, 95% CI = 0.23, 0.57), marital status (OR = 0.51, 95% CI = 0.33, 0.80), having never received addiction treatment (OR = 0.39, 95% CI = 0.25, 0.61), not having discussed with PE/CE (OR = 0.55, 95% CI = 0.32, 0.96) and not having knowledge of ART (OR = 0.21, 95% CI = 0.13,0.33) (Tables 4 and 5). In addition to this, not having had sex with a FSW in the past year was also protective, however had a statistically insignificant confidence interval.

**Table 4:** Socio-demographic determinants of HIV Prevalence among male injecting drug users, Pokhara Valley (Nepal)

	OR (95% CI)					
	2007	2009	2011	2015	2017	2007–2017
Socio-demographic Factors						
*Age*						
Below Median Age	1.00	1.00	1.00	–	1.00	1.00
Above Median Age	4.47 (1.73–11.53)	4.41 (1.40–13.94)	5.43 (1.51–19.50)	–	9.47 (2.12–42.48)	5.75 (3.19–10.36)
*Educational achievement Status*						
Primary or lower	1.00	1.00	1.00	1.00	1.00	1.00
Secondary or higher	0.35 (0.16–0.80)	0.21 (0.08–0.59)	0.46 (0.15–1.38)	0.43 (0.10–1.76)	0.52 (0.16–1.66)	0.36 (0.23–0.57)
*Marital Status*						
Not married	1.00	1.00	1.00	1.00	1.00	1.00
Married	1.15 (0.51–2.71)	1.17 (0.42–3.28)	1.50 (0.53–4.26)	18.62 (2.28–151.90)	3.26 (1.17–9.08)	1.94 (1.25–3.02)
*Living with sexual partner situation*						
Yes	1.00	1.00	1.00	1.00	1.00	1.00
No	1.12 (0.47–2.68)	1 (0.34–2.93)	0.63 (0.22–1.78)	0.24 (0.06–0.93)	0.49 (0.17–1.46)	0.70 (0.44–1.13)

Logistic regression models were estimated using the combined datasets from 2007 to 2017 with year being adjusted for as a confounder. The models examined socio-demographic factors and whether these acted as determinants of HIV status over the period of 2007–2017. The 2003 and 2005 datasets were excluded from this analysis as the inflated prevalence of HIV in those years would have skewed the results. Some variables presented in Tables 1-3 were excluded from models due to small case numbers and unstable estimates

**Table 5:** Knowledge, behavioural and health service determinants of HIV among male injecting drug users, Pokhara Valley (Nepal)

	OR (95% CI)					
	2007	2009	2011	2015	2017	2007–2017
*Recent use of unsterilised needles*						
Yes	1.00	1.00	1.00	–	–	1.00
No	0.27 (0.11–0.67)	0.63 (0.23–1.68)	0.24 (0.07–0.79)	–	–	0.45 (0.24–0.83)
*Avoids needle sharing behaviour*						
No	1.00	1.00	1.00	–	–	1.00
Yes	0.13 (0.04–0.48)	0.38 (0.05–3.03)	0.07 (0.01–0.75)	–	–	0.54 (0.20–1.41)
*Age at first injection*						
Below median	1.00	1.00	1.00	1.00	1.00	1.00
Above median	0.52 (0.22–1.22)	0.73 (0.25–1.92)	0.23 (0.06–0.83)	4.78 (0.97–23.49)	3.81 (1.21–11.98)	1.06 (0.68–1.66)
*Received addiction treatment*						
Yes	1.00	1.00	1.00	1.00	1.00	1.00
No	0.35 (0.15–0.81)	0.74 (0.27–1.97)	0.47 (0.16–1.40)	0.23 (0.06–0.93)	0.26 (0.10–0.70)	0.39 (0.25–0.61)
*HIV test*						
Yes	1.00	1.00	1.00	1.00	–	1.00
No	1.36 (0.60–3.09)	1.78 (0.65–4.85)	0.34 (0.07–1.52)	0.35 (0.07–1.74)	–	0.74 (0.46–1.21)
*Condom use (past 12 months)*						
Always	1.00	1.00	–	1.00	–	1.00
Never	0.46 (0.07–3.12)	0.27 (0.02–3.59)	–	0.08 (0.01–1.45)	–	1.35 (0.57–3.20)
*Duration of ID use*						
Below median	1.00	1.00	1.00	1.00	1.00	1.00
Above median	5.37 (2.08–13.85)	3.63 (1.30–10.15)	9.92 (2.75–35.75)	14.21 (1.74–115.87)	9.23 (2.07–41.25)	6.94 (3.91–12.31)
*Use of FSW (past 12 months)*						
Yes	1.00	1.00	1.00	1.00	1.00	1.00
No	1.09 (0.48–2.45)	2.43 (0.67–8.86)	0.90 (0.31–2.59)	0.51 (0.13–1.96)	0.45 (0.16–1.27)	0.93 (0.58–1.48)
*Alcohol use (past 12 months)*						
Yes	1.00	1.00	1.00	1.00	1.00	1.00
No	0.82 (0.27–2.46)	0.62 (0.18–2.07)	0.60 (0.14–2.51)	0.69 (0.09–5.05)	1.83 (0.37–8.87)	0.80 (0.45–1.41)
*Discussed with PE/OE/CM/CE*						
Yes	1.00	1.00		1.00	1.00	1.00
No	1.32 (0.57–3.04)	0.79 (0.17–3.59)		0.20 (0.05–0.77)	0.39 (0.15–1.06)	0.55 (0.32–0.96)
*Knowledge of where to get ART*						
Yes	1.00	1.00	1.00	–	1.00	1.00
No	0.27 (0.12–0.62)	0.49 (0.17–1.39)	0.39 (0.14–1.08)	–	0.02 (0.01–0.16)	0.21 (0.13–0.33)

*ID* Injection drugs, *FSW* Female sex worker, *PE/OE/CM/CE* Peer Educators, Outreach Educators, Community Motivators/Mobilisers, Community Educators, Antiretroviral therapy.

Logistic regression models were run on the combined datasets from 2007 to 2017 with year being adjusted for as a confounder. The models examined socio-demographic factors and whether these acted as determinants of HIV status over the period of 2007–2017. The 2003 and 2005 datasets were excluded from this analysis as the inflated prevalence of HIV in those years would have skewed the results. Some variables presented in Tables 1-3 were excluded from models due to small case numbers and unstable estimates. Models are adjusted for age, education level and marital status

A higher risk of HIV was associated with being above the median age of 24 (OR = 5.75, 95% CI = 3.19, 10.36) and having injected drugs for more than the median duration of 4 years (OR = 6.94, 95% CI = 3.91, 12.31) (Table 5.). Not using a condom with an FSW and first injecting later than the median age of 20 years old were also associated with higher risk of HIV prevalence, although differences were not statistically significant (Table 5.).

## Discussion

The overall prevalence of HIV among male PWIDs in the Pokhara valley varied across the study period examined in this study. In 2003 and 2005, the prevalence was recorded as 22.0 and 21.7%, before declining to 8.7% in 2007 and 5.7% in 2009. There was an increase in 2011 to 11.3%, before a marked decline in 2015 to 2.6%. In the most recent survey at the time of this study, the prevalence recorded for 2017 was 4.9%, indicating that although lower than the 2011 measurement, the prevalence of HIV among male PWIDs increased in the most recent period.

The findings in this study indicate that the main behavioural determinants of HIV among male PWIDs in the Pokhara valley are use of unsterilized needles, a lower level of education and high alcohol intake. Low alcohol use and not having sexual intercourse with female sex workers in the last year were both associated with a lower risk of HIV, although the association was statistically insignificant. It was found that having an education level of secondary school or higher, not being married, having never received addiction treatment, not having discussed with PE/CE and not having knowledge of ART were all associated with lower risk of HIV.

Findings also indicate that the number of IDUs who had ever received treatment for their addiction has decreased since 2007. However, daily alcohol consumption and use of female sex workers decreased over the study period, which occurred contemporaneously with the decline in HIV prevalence from 2007 onwards. In the most recent survey, the decrease in alcohol consumption is potentially due to changes in government regulations on alcohol [23]. Analysis of the prevalence of different behaviours indicated that frequency of the majority of needle sharing behaviours also decreased. Education status was not consistently significant in either the unadjusted or adjusted models, although individual years produced statistically significant ORs. One study based on drug use in third-year medical students in Kathmandu noted that most respondents reported that they only began using drugs after admission to medical school [24]. This potentially indicates that drug use is closely associated with education levels.

Younger age was found to be associated with a lower risk of HIV in all models, which is consistent with previous findings [25, 26]. There are slight differences in the magnitude of the observed association, however this may have been due to the differences in classification when creating a dichotomous age category. Similarly, not being married was associated with lower risk of HIV in several of the models from this study.

Recent use of unsterilized injection equipment was predictably a risk factor for HIV status in the 2007–2017 models, although was not consistently statistically significant. This may be due to a variety of factors, such as difference in cleaning methods or the effect of the demographic confounders being adjusted for. Unsterilized or contaminated equipment has been shown to be a risk factor in multiple different settings [4, 8, 27, 28]. This problem is likely to be exacerbated by the low coverage of programs aimed at providing PWIDs with clean injecting equipment. Data from 2016 shows that the current rate of distribution of clean needles is far lower than the recommended standard of at least 200 per PWID per year [29].

In general, HIV knowledge was high among the participants of the surveys. Most of the participants knew someone with HIV/AIDS, and most had what was determined by the DHS standards as a “comprehensive knowledge of HIV”. The number of individuals with comprehensive knowledge increased from 2015 to 2017, suggesting better engagement in HIV education services in male PWIDs. However, the high risk associated with having experienced addiction treatment and using PE suggests that although the knowledge of HIV was relatively high, health programs may have been accessed insufficiently before the individual knew their HIV status. It should also be noted that coverage of these healthcare programs in Nepal is limited, which may be the reason for lower rates of accessing these programs. Issues with service access outside of major population centres and stigma within the general population may have limited the effectiveness of addiction interventions. Reducing stigma around drug use and addiction, as well as HIV, could be a potential strategy to increase access to these programs.

Conversely, knowledge of where to receive antiretroviral therapy reached the lowest number in 2017. Compared to other developing countries, the level of knowledge of HIV in Nepal is encouraging. Data gathered from the DHS (Demographic and Health Surveys) and AIDS Indicator Surveys (AIS) in 33 sub-Saharan countries showed only minimal increases in the level and spread of HIV knowledge between 2003 and 2015 [30].

There are several potential limitations with this study. Most of these relate to the cross-sectional nature of the data, conducted at multiple time points which opens up the potential for small shifts in methodology to affect consistency in measurement over time. In addition to this, some of the data from the 2017 set was missing (including data on knowing someone with HIV), meaning that some risk factors were unable to be properly investigated in this year.

It is also difficult to determine the role of changes in legislation or other HIV specific interventions across the timespan of the IBBS surveys. The difficulties in utilising the findings from IBBS surveys has been noted in the past, with several reasons provided for why the interpretation and implementation of findings is complicated [31]. The difficulties mentioned are also likely to impact the results of the surveys, such as issues with sampling consistency. Some sources of measurement bias are likely to be present in the surveys, as they were conducted by different people across a relatively large time. Additionally, the survey results relied heavily upon the self-reporting of the participants, potentially creating inaccuracies. Selection bias is also possible, as volunteerism means that the IDUs selected for the survey may not have been representative of all IDUs in Pokhara.

Due to the cross-sectional study design, temporality and causality are also difficult to establish. Although the answers for variables may indicate causality, it cannot be ascertained which of the variables came first.

However, there are strengths to the study. For example, analysing the changing risk of different behaviours over time is helpful in determining if health programs are acting effectively in reducing HIV prevalence. HIV diagnoses were not reliant on self-reporting, but were based on biological samples, reducing the likelihood of reporting bias. In addition to this, knowing which behavioural and demographic risk factors are becoming more prevalent in recent years is valuable information for health service programs. Targeted HIV prevention programs have provided encouraging results, both in Nepal [32] and other developing countries [33–35]. Strengthening and tailoring the current HIV programs to better suit the individuals who need them should be a priority moving forward.

Increasing both the knowledge of and access to antiretroviral therapy is also a potential avenue of reducing the spread of HIV, as it has been shown that ART is one of the most effective mechanisms of controlling HIV [36]. The issues with ART adherence and access in Nepal have been noted previously [37]. In the most recent government strategy document related to HIV control, the National HIV Strategy 2016–2021 [29] targets 90% retention for individuals diagnosed with HIV on ART, while also aiming to identify, test and correctly diagnose 90% of the key populations [29]. The results found in this paper appear to show that the numbers of IDUs who have been tested for HIV, while increasing, remain below the 90% target testing rate. Additionally, the numbers of HIV positive individuals in this study who know where and how to get ART were far lower than the target, as only 16% of those with HIV and only 12% of IDUs overall knew where to receive this treatment.

## Conclusion

As Pokhara represents one of the largest population centres in Nepal and a hub for tourism, the findings are somewhat representative of many other large population centres within the country. The prevalence of HIV in male PWIDs in Pokhara has been variable, but overall has reduced in recent years reaching 2.6% before increasing in 2017 to 4.9%. This study has identified several important socio-demographic and modifiable behavioural risk factors associated with trends in HIV prevalence among male PWIDs in Nepal to inform current population health policy strategies and responses. Based on the levels of knowledge of the male PWIDs, addiction treatment and HIV education programs need to be strengthened in future intervention strategies.

## Supplementary Information

**Additional file 1: Table S1.** Participant characteristics of the Integrated Biological Behavioural Surveys, Pokhara Valley (Nepal).

## Data Availability

The datasets used and/or analysed during the current study are available from the corresponding author on reasonable request. CMDN maintain the individual datasets from IBBS studies, with information on each IBBS study available here: http://library.nhrc.gov.np:8080/nhrc/handle/123456789/18/search

## Abbreviations

AIDS: Acquired Immune Deficiency Syndrome
AIS: AIDS Indicator Surveys
ART: Antiretroviral Therapy
DHS: Demographic and Health Surveys
FSW: Female Sex Worker
HIV: Human Immunodeficiency Virus
IDU: Intravenous Drug User
IBBS: Integrated Biological Behavioural Survey
PE/CE: Peer Educator/Community Educator
PE/OE/CM/CE: Peer Educators/Outreach Educators/Community Motivators/ Community Educators
PWID: People Who Inject Drugs
