# Supplementary material for: Trends and determinants of HIV transmission among men who inject drugs in the Pokhara Valley, Nepal: analysis of cross-sectional studies
Source: BMC Public Health. 2021 Feb 2;21:269. doi: 10.1186/s12889-021-10331-9 (PMC7856790; doi:10.1186/s12889-021-10331-9)
Supplement: Supplementary file 1 — Additional file 1: Table S1. Participant characteristics of the Integrated Biological Behavioural Surveys, Pokhara Valley (Nepal). [file 12889_2021_10331_MOESM1_ESM.docx]

**Supplementary Table 1: Participant characteristics of the** **Integrated Biological Behavioural Surveys, Pokhara Valley (Nepal).**

|  | **2003** | **2005** | **2007** | **2009** | **2011** | **2015** | **2017** |
| --- | --- | --- | --- | --- | --- | --- | --- |
|  | n (%) | n (%) | n (%) | n (%) | n (%) | n (%) | n (%) |
| **Socio-demographic Characteristics** | | |  |  |  |  |  |
| **Age** |  |  |  |  |  |  |  |
| Below median age | 161 (53.7) | 164 (54.7) | 163 (54.3) | 167 (55.7) | 186 (53.9) | 180 (52.2) | 185 (53.6) |
| above median age | 139 (46.3) | 136 (45.3) | 137 (45.7) | 133 (44.3) | 159 (46.1) | 165 (47.8) | 160 (46.4) |
| **Education Level** |  |  |  |  |  |  |  |
| Primary or lower | 100 (33.3) | 114 (38) | 94 (31.3) | 90 (30) | 62 (18) | 62 (18) | 49 (14.2) |
| Secondary or Higher | 200 (66.7) | 186 (62) | 206 (68.7) | 210 (70) | 283 (82) | 283 (82) | 296 (83.8) |
| **Marital status** |  |  |  |  |  |  |  |
| Not married | 218 (72.7) | 213 (71) | 195 (65) | 204 (68) | 200 (66.7) | 236 (68.4) | 216 (62.6) |
| Married | 82 (27.8) | 87 (29) | 105 (35) | 96 (32) | 100 (33.3) | 109 (31.6) | 129 (37.4) |
| **Living with sexual partner** | |  |  |  |  |  |  |
| Yes | 80 (26.7) | 85 (28.3) | 99 (33) | 88 (29.3) | 96 (27.8) | 83 (24.1) | 61 (17.7) |
| No | 220 (73.3) | 215 (71.7) | 201 (67) | 212 (70.7) | 249 (72.2) | 262 (75.9) | 284 (82.3) |
| **Drug Injecting Behaviours** | | |  |  |  |  |  |
| **Injected on Previous day** | | |  |  |  |  |  |
| Yes | 243 (81) | 145 (48.3) | 236 (78.7) | 237 (78.7) | 206 (59.7) | 41 (11.9) | 152 (44.1) |
| No | 57 (19) | 155 (51.7) | 64 (21.3) | 65 (21.3) | 139 (40.3) | 304 (88.1) | 193 (55.9) |
| **Recently used unsterilised needles** | | |  |  |  |  |  |
| Yes | - | - | 37 (12.3) | 20 (6.7) | 28 (8.1) | 6 (2.4) | 34 (10.4) |
| No | - | - | 263 (87.7) | 280 (93.3) | 317 (91.9) | 244 (97.6) | 292 (89.6) |
| **Injected with used syringe in the past week** | | |  |  |  |  |  |
| Yes | 63 (21) | 44 (14.7) | 25 (8.3) | 16 (5.3) | 8 (2.3) | 1 (0.3) | N/A |
| No | 229 (76.3) | 195 (65) | 267 (89) | 276 (92) | 307 (89) | 139 (40.3) | N/A |
| Not applicable | 8 (2.7) | 61 (20.3) | 8 (2.7) | 8 (2.7) | 30 (8.7) | 205 (59.4) | N/A |
| **Gave needle to someone else after use in last week** | | | |  |  |  |  |
| Yes | 66 (22) | 38 (12.7) | 16 (5.3) | 9 (3) | 8 (2.3) | 3 (0.9) | 11 (3.2) |
| No | 226 (75.3) | 201 (67) | 276 (92) | 283 (94.3) | 307 (89) | 137 (39.7) | 333 (96.5) |
| Not applicable | 8 (2.7) | 61 (20.3) | 8 (2.7) | 8 (2.7) | 30 (8.7) | 205 (59.4) | 1 (0.3) |
| **Used a pre-filled syringe in past week** | | | |  |  |  |  |
| Yes | 23 (7.6) | 25 (8.3) | 17 (5.7) | 41 (13.6) | 8 (2.3) | 3 (0.9) | 27 (7.8) |
| No | 269 (89.7) | 213 (71) | 275 (91.6) | 251 (83.7) | 307 (89) | 137 (39.7) | 316 (91.6) |
| Not applicable | 8 (2.7) | 53 (17.7) | 8 (2.7) | 8 (2.7) | 30 (8.7) | 205 (59.4) | 2 (0.6) |
| **Know where to get clean needles** | | |  |  |  |  |  |
| Yes | 300 (100) | 294 (98) | 295 (98.3) | 299 (99.7) | 344 (99.7) | 310 (89.9) | 301 (87.2) |
| No | 0 (0) | 6 (2) | 5 (1.7) | 1 (0.3) | 1 (0.3) | 35 (10.1) | 44 (12.8) |
| **Average age at first injection (years)** | | |  |  |  |  |  |
| Above median | 169 (56.3) | 152 (50.7) | 153 (51) | 169 (56.3) | 178 (51.6) | 196 (56.8) | 181 (52.5) |
| Below median | 131 (45.7) | 148 (49.3) | 147 (49) | 131 (45.7) | 167 (48.4) | 149 (43.2) | 164 (47.5) |
| **Health Factors/Services** | | |  |  |  |  |  |
| **Received addiction treatment** | | |  |  |  |  |  |
| Yes | 91 (30.3) | 132 (44) | 115 (38.3) | 120 (40) | 179 (51.9) | 111 (32.2) | 83 (24.1) |
| No | 209 (69.7) | 168 (56) | 185 (62.7) | 180 (60) | 166 (48.1) | 234 (67.8) | 262 (75.9) |
| **HIV test** |  |  |  |  |  |  |  |
| Yes | 80 (26.7) | 193 (64.3) | 193 (64.3) | 213 (71) | 245 (71) | 193 (55.9) | 218 (63.2) |
| No | 220 (73.3) | 107 (35.7) | 107 (35.7) | 87 (29) | 100 (29) | 152 (44.1) | 127 (36.8) |
| **Condom Use** |  |  |  |  |  |  |  |
| Always | 8 (2.7) | 11 (3.7) | 14 (4.7) | 6 (2) | 5 (1.4) | 6 (1.7) | N/A |
| Sometimes | 32 (10.7) | 40 (13.3) | 43 (14.3) | 51 (17) | 51 (14.8) | 31 (9) | N/A |
| Never | 46 (15.3) | 38 (12.7) | 42 (14) | 39 (13) | 42 (12.2) | 67 (19.4) | N/A |
| Not Applicable | 214 (71.3) | 211 (70.3) | 201 (67) | 204 (68) | 247 (71.6) | 241 (69.9) | N/A |
| **Duration of ID use** |  |  |  |  |  |  |  |
| Below median duration | 196 (65.3) | 176 (58.7) | 175 (58.3) | 194 (64.7) | 232 (67.2) | 216 (62.6) | 183 (53) |
| Above median duration | 104 (34.7) | 124 (41.3) | 125 (41.7) | 106 (35.3) | 113 (32.8) | 129 (37.4) | 162 (47) |
| **Use of FSW (past 12 mths)** | |  |  |  |  |  |  |
| Yes | 99 (35.4) | 121 (42) | 139 (48.1) | 105 (38.5) | 126 (37.5) | 96 (29.5) | 69 (20.5) |
| No | 181 (64.6) | 167 (58) | 150 (51.9) | 168 (61.5) | 210 (62.5) | 229 (70.5) | 267 (79.5) |
| **Drinking Alcohol** |  |  |  |  |  |  |  |
| Every day | 127 (42.3) | 170 (58.2) | 106 (35.3) | 101 (33.7) | 88 (25.7) | 59 (17.1) | 56 (16.2) |
| Sometimes | 126 (42) | 96 (32.9) | 141 (47) | 136 (45.3) | 186 (54.4) | 201 (58.3) | 163 (47.2) |
| Never | 47 (15.7) | 26 (8.9) | 53 (17.7) | 63 (21) | 68 (19.9) | 85 (24.6) | 126 (36.5) |
| **Health Knowledge/Stigma of HIV** | | |  |  |  |  |  |
| **Know anyone with HIV/AIDS** | | |  |  |  |  |  |
| Yes | 194 (64.7) | 240 (80) | 231 (77) | 223 (74.3) | 245 (71) | 229 (66.4) | N/A |
| No | 97 (32.3) | 49 (16.3) | 69 (23) | 77 (25.7) | 100 (29) | 116 (33.6) | N/A |
| Unsure | 9 (3) | 11 (3.7) | 0 (0) | 0 (0) | 0 (0) | 0 (0) | 0 (0) |
| **Discussed with PE/OE/CM/CE** | | |  |  |  |  |  |
| Yes | - | - | 202 (67.3) | 257 (85.7) | 295 (85.5) | 72 (20.9) | 93 (27) |
| No | - | - | 98 (32.7) | 43 (14.3) | 50 (14.5) | 273 (79.1) | 252 (73) |
| **Knowledge of ART** |  |  |  |  |  |  |  |
| Yes | - | - | 89 (29.7) | 66 (22) | 101 (29.3) | 127 (37.8) | 98 (28.5) |
| No | - | - | 211 (70.3) | 234 (78) | 244 (80.7) | 209 (62.2) | 246 (71.5) |

FSW - Female Sex Worker

HIV/AIDS - Human Immunodeficiency virus/ Acquired Immune Deficiency Syndrome

PE/OE/CM/CE - Peer Educators, Outreach Educators, Community Motivators/Mobilisers, Community Educators

ART - Antiretroviral therapy
